# The Impact of Nonmotor Symptoms on Health-Related Quality of Life in Parkinson’s Disease: A Network Analysis Approach

**DOI:** 10.3390/jcm12072573

**Published:** 2023-03-29

**Authors:** Konstantin G. Heimrich, Aline Schönenberg, Diego Santos-García, Pablo Mir, COPPADIS Study Group, Tino Prell

**Affiliations:** 1 Department of Neurology, Jena University Hospital, Am Klinikum 1, 07747 Jena, Germany; 2 Department of Geriatrics, Halle University Hospital, Ernst-Grube-Straße 40, 06120 Halle, Germany; 3 Department of Neurology, CHUAC (Complejo Hospitalario Universitario de A Coruña), c/As Xubias 84, 15006 A Coruña, Spain; 4 Unidad de Trastornos del Movimiento, Servicio de Neurología y Neurofisiología Clínica, Instituto de Biomedicina de Sevilla, Hospital Universitario Virgen del Rocío/CSIC/Universidad de Sevilla, 41013 Seville, Spain; 5 Centro de Investigación Biomédica en Red Sobre Enfermedades Neurodegenerativas (CIBERNED), 28031 Madrid, Spain; 6 Fundación Española de Ayuda a la Investigación en Enfermedades Neurodegenerativas y/o de Origen Genético, Calle Antonio J de Sucre 1A, 15179 Oleiros, Spain

**Keywords:** Parkinson disease, nonmotor symptoms, quality of life, cognition, depression, fatigue, hyperhidrosis, disorders of excessive somnolence, network analysis, NMSS, PDQ-39

## Abstract

Nonmotor symptoms negatively affect health-related quality of life (HRQoL) in patients with Parkinson’s disease (PD). However, it is unknown which nonmotor symptoms are most commonly associated with HRQoL. Considering the complex interacting network of various nonmotor symptoms and HRQoL, this study aimed to reveal the network structure, explained HRQoL variance, and identify the nonmotor symptoms that primarily affect HRQoL. We included 689 patients with PD from the Cohort of Patients with Parkinson’s Disease in Spain (COPPADIS) study who were rated on the Nonmotor Symptoms Scale in Parkinson’s disease (NMSS) and the Parkinson´s Disease Questionnaire 39 (PDQ-39) at baseline. Network analyses were performed for the 30 items of the NMSS and both the PDQ-39 summary index and eight subscales. The nodewise predictability, edge weights, strength centrality, and bridge strength were determined. In PD, nonmotor symptoms are closely associated with the mobility, emotional well-being, cognition, and bodily discomfort subscales of the PDQ-39. The most influential nonmotor symptoms were found to be fatigue, feeling sad, hyperhidrosis, impaired concentration, and daytime sleepiness. Further research is needed to confirm whether influencing these non-motor symptoms can improve HRQoL.

## 1. Introduction

Parkinson’s disease (PD) is a progressive, multisystem neurodegenerative disorder characterized by motor and nonmotor symptoms [1]. Owing to the progressive nature of the disease, health-related quality of life (HRQoL) is an essential focus when treating PD. Numerous studies have shown the contribution of nonmotor symptoms to the deterioration of patients’ quality of life [2,3,4,5,6,7,8,9,10,11]. However, nonmotor symptoms are often poorly recognized [2].

HRQoL is a multidimensional concept that is commonly used to examine the impact of health status on the quality of life. It usually includes physical, mental, and social domains of health [12]. The Parkinson´s Disease Questionnaire 39 (PDQ-39) is the most thoroughly tested and frequently applied questionnaire [13]. It is a disease-specific and self-rated questionnaire that detects minor changes in HRQoL [14,15]. The 39 items included in the PDQ-39 are grouped into eight domains and their respective subscales, the mean value of which determines the summary index. However, as mentioned above, HRQoL is a multidimensional concept, and using the summary index of the PDQ-39 instead of the eight subscales does not adequately account for its complexity [16]. Moreover, patients with limitations in the physical domain of HRQoL are expected to require different therapies than those with limitations in the emotional or social domains. Therefore, consideration of the PDQ-39 subscales provides more specific information.

The Nonmotor Symptoms Scale in Parkinson’s Disease (NMSS) is frequently used to comprehensively assess a range of nonmotor symptoms in patients with PD [17,18,19]. The NMSS consists of 30 items. Each item describes a different nonmotor symptom and considers its severity and frequency. Therefore, the NMSS is a suitable tool for detecting and quantifying nonmotor symptoms in patients with PD. Accordingly, many original studies have used the scale as a clinical outcome measure of nonmotor symptoms [19]. However, owing to the variety of nonmotor symptoms, the total score or domain structure of the NMSS is usually used for statistical analysis. Taking into account the known shortcomings of the NMSS and the limited internal consistency of the domain structure [17,18,20], the consideration of nonmotor symptoms on a single-item level seems advantageous. However, this generally requires more patients, which limits its applicability.

Although previous studies have examined the association between the NMSS and PDQ-39 [7,9,11], the particular symptoms that are most commonly associated with decreased HRQoL have not been clarified, considering the complex interacting network of all nonmotor symptoms. However, due to the variety of nonmotor symptoms, it is important to uncover how these symptoms are linked to HRQoL and to reveal which nonmotor symptoms impact HRQoL. Therefore, we considered both the PDQ-39 summary index and the eight PDQ-39 subscales. Identifying the most important factors that determine HRQoL would allow the prioritization of interventions [21], providing the basis for the improved holistic treatment of patients with PD. 

Network analysis is an appropriate tool for gaining these insights, considering all relevant associations between different variables. Accordingly, the present study aimed to reveal (1) the structure of the complex interacting networks of various nonmotor symptoms and HRQoL in PD, (2) the proportion of the HRQoL variance that can be explained by nonmotor symptoms, and (3) which nonmotor symptoms primarily affect HRQoL. This knowledge is crucial as it represents a promising way to improve HRQoL in patients with PD.

## 2. Materials and Methods

### 2.1. Study Design

Data were extracted from the Cohort of Patients with Parkinson’s Disease in Spain (COPPADIS) study, a national, multicenter, longitudinal study [22]. PD patients aged between 30 and 75 years without dementia were initially recruited from 35 centers in Spain from January 2016 to November 2017. Detailed information on the study design is provided in the COPPADIS study protocol [22].

### 2.2. Participants

In this study, we included patients with PD whose NMSS and PDQ-39 scores were obtained at the baseline evaluation, resulting in a sample of 689 patients.

### 2.3. Variables

The NMSS was used to assess nonmotor symptoms. The scale comprises 30 items that describe different nonmotor symptoms experienced during the previous month. The score for each item is calculated by multiplying the severity (0 = none, 1 = mild, 2 = moderate, 3 = severe) and frequency (1 = rarely, 2 = often, 3 = frequent, 4 = very frequent), ranging from 0 to 12 points. The total NMSS score ranges from 0 to 360 points. Theoretically, items are assigned to nine different domains: cardiovascular (domain 1; items 1 and 2), sleep/fatigue (domain 2; items 3, 4, 5, and 6), mood/cognition (domain 3; items 7, 8, 9, 10, 11, and 12), perceptual problems (domain 4; items 13, 14, and 15), attention/memory (domain 5; items 16, 17, and 18), the gastrointestinal tract (domain 6; items 19, 20, and 21), urinary (domain 7; items 22, 23, and 24), sexual function (domain 8; items 25 and 26), and miscellaneous (domain 9; items 27, 28, 29, and 30) [17]. However, for the network analyses, we considered all 30 items.

To assess HRQoL, the subscales and summary indices of the PDQ-39 were considered. The PDQ-39 is a self-rated questionnaire consisting of 39 items divided into eight subscales: mobility (MOB, 10 items), activities of daily living (ADL, 6 items), emotional well-being (EMO, 6 items), stigma (STI, 4 items), social support (SOC, 3 items), cognition (COG, 4 items), communication (COM, 3 items), and bodily discomfort (BOD, 3 items). There are five possible answers for each item: never, occasionally, sometimes, often, and always. Each subscale is converted into a score ranging from 0 to 100 (higher values indicate worse HRQoL). The PDQ-39 summary index is calculated as the mean of the eight subscales and may represent a single value for assessing patients’ overall HRQoL. Details of the scoring system for the PDQ-39 can be found in the PDQ user manual [23]. 

In addition, the following variables were extracted: patient age, sex, Hoehn and Yahr stage [24], and Unified Parkinson’s Disease Rating Scale (UPDRS) part III [25].

### 2.4. Statistical Analysis

For descriptive statistics, normality was tested using the Shapiro–Wilk test. The results are reported as numbers and percentages for categorical variables and the median and interquartile range (IQR) for non-normally distributed continuous variables. The statistical significance for all tests was set at *p* < 0.05 (two-tailed).

Network analyses based on partial correlations were conducted to explore the associations between the 30 NMSS items and HRQoL. In this network approach, the individual nonmotor symptoms and HRQoL measures are seen as complex interacting systems. Therefore, partial correlations refer to associations between two random variables, taking into account other confounding variables related to both variables of interest. Accordingly, the overall pattern of connections is considered to understand interactions, rather than looking at individual correlations that do not take into account whether there is another variable causing that relationship. To precisely assess the influence of nonmotor symptoms on HRQoL, the latter was considered first as the PDQ-39 summary index; second, each of the eight PDQ-39 subscales were considered separately; and third, all subscales were included. This approach was used to identify individual nonmotor symptoms and HRQoL measures.

In general, networks contain two fundamental components: nodes, representing the variables entered into the model, and edges, displaying the correlations between the nodes. Edge thickness reflects the intensity of the connection. Moreover, every node is positioned using the Fruchterman–Reingold algorithm based on the strength of the connections between nodes using pseudorandom numbers [26]. However, instead of relying on simple correlations, a regularization technique, which takes the model complexity into account, is frequently used to prevent overfitting of the partial correlation network structure by reducing the number of spurious correlations between variables [27]. In this study, we used the extended Bayesian information criterion (EBIC) [28,29] with the least absolute shrinkage and selection operator (LASSO) [30]. For more sensitive and specific network analysis, the EBICglasso tuning parameter was set to 0.5, resulting in a sparse network. Nonparanormal transformation (npn) was conducted to generate a normal distribution of non-normally distributed data. 

In addition to the layout of the nodes and their edges, centrality measures can be used to assess the variables’ influences and their connections statistically. The strength centrality measure was determined for each node using standardized values. Therefore, the strength of a node corresponds to the sum of the absolute edge weights associated with that node [31] and, accordingly, describes the direct connections to other nodes [31,32,33]. In clinical practice, a node with a high-strength centrality measure can be a potential therapeutic target, because a change in the value of this node can rapidly influence other nodes within the network. In addition, the nodewise predictability was determined to quantify how well a given node (i.e., PDQ) can be predicted by all other nodes connected in the network (i.e., associated nonmotor symptoms) [34]. The determined explained variance R2 can range from 0 to 1, and values ≥ 0.13 are considered moderate and values ≥ 0.26 are considered high [35]. 

To identify nonmotor symptoms with the greatest impacts on the eight PDQ-39 subscales, the bridge strength was calculated. The bridge strength is defined as the sum of the absolute values of all edges that exist between a node of a community (i.e., a nonmotor symptom) and all nodes from another community (i.e., PDQ-39 subscales) [36]. Accordingly, a nonmotor symptom with a high bridge strength substantially impacts all PDQ-39 subscales compared with other nonmotor symptoms within the network. 

To demonstrate the network’s stability, the correlation stability (CS) coefficient was estimated using a case-dropping bootstrap procedure (number of bootstraps = 1000). The CS coefficient quantifies the proportion of cases that can be dropped to retain a correlation with an original strength of at least 0.7 in at least 95% of the samples [33]. To confirm that the network structure was stable, the CS coefficient should preferably exceed 0.5 [33]. 

Statistical analyses were performed using SPSS (IBM SPSS Statistics 27, IBM, Armonk, NY, USA), R (version 4.2.1, R Foundation for Statistical Computing, Vienna, Austria), and JASP (version 0.15, JASP Team, Amsterdam, The Netherlands) software.

## 3. Results

### 3.1. Descriptive Analysis

Of the 689 patients with PD, 414 (60.1%) were male, and 275 (39.9%) were female. The median patient age was 64 years (IQR = 57–70 years). Most patients presented with a disease stage of bilateral involvement (Hoehn and Yahr stage ≥ 2) and moderate motor impairment (median UPDRS III: 21 points, IQR = 14–30). According to the PDQ-39, the patients rated their HRQoL with a median summary index of 12.8 points (IQR = 7.7–24.4). Patients assessed their HRQoL as particularly poor in terms of the bodily discomfort (median value: 25.0, IQR = 8.3–41.6), cognition (median value: 18.7, IQR = 6.3–31.2), and emotional well-being subscales (median value: 16.6, IQR = 4.2–33.3). Patients reported nonmotor symptoms with a median NMSS total score of 35 points (IQR = 19–61). The descriptive statistics of the study population are presented in Table 1. 

### 3.2. Network Structure

#### 3.2.1. PDQ-39 Summary Index

**Figure 1:**
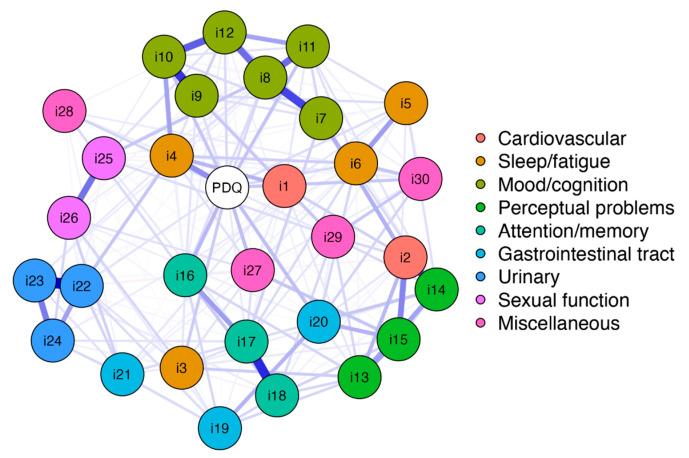
Network structure of the Nonmotor Symptoms Scale in Parkinson’s disease (NMSS) and Parkinson’s Disease Questionnaire 39 (PDQ-39) summary index. The node *PDQ* displays the PDQ-39 summary index, and nodes *i1–i30* display the items included in the NMSS. The thickness of the edges indicates the strength of the correlations between these nodes. Color coding represents the assignment of the items to the nine domains of the NMSS: cardiovascular (domain 1; items 1 and 2); sleep/fatigue (domain 2; items 3, 4, 5, and 6); mood/cognition (domain 3; items 7, 8, 9, 10, 11, and 12); perceptual problems (domain 4; items 13, 14, and 15); attention/memory (domain 5; items 16, 17, and 18); gastrointestinal tract (domain 6; items 19, 20, and 21); urinary (domain 7; items 22, 23, and 24); sexual function (domain 8; items 25 and 26); and miscellaneous (domain 9; items 27, 28, 29, and 30). Item 1: light headedness; item 2: fainting; item 3: daytime sleepiness; item 4: fatigue; item 5: sleep initiation; item 6: restless legs; item 7: loss of interest; item 8: lack of motivation; item 9: feeling nervous; item 10: feeling sad; item 11: flat mood; item 12: anhedonia; item 13: hallucinations; item 14: delusions; item 15: diplopia; item 16: concentration; item 17: forgetfulness; item 18: forget to do things; item 19: sialorrhea; item 20: dysphagia; item 21: constipation; item 22: urgency; item 23: frequency; item 24: nocturia; item 25: interest; item 26: problems having sex; item 27: pain; item 28: taste/smell; item 29: weight change; and item 30: hyperhidrosis.

The network structure between nonmotor symptoms and the PDQ-39 summary index is shown in Figure 1. The white node displays the PDQ-39 summary index (*PDQ*), and the colored nodes display the NMSS items (i1–i30). Therefore, the color assignment of the nodes corresponds to the distribution of items in the domain structure of the NMSS.

On a global level, the network analysis revealed a well-connected network (229 of 465 nonzero edges). None of the 31 nodes were separated entirely. The pre-existing structure of the NMSS based on the nine domains cannot be visually delimited owing to the numerous associations of items from different domains. While items of all the NMSS domains were associated with *PDQ*, the edge between item 4 (*fatigue*) and *PDQ* had the highest weight (edge weight 0.184, as shown in Table S1).

**Figure 2:**
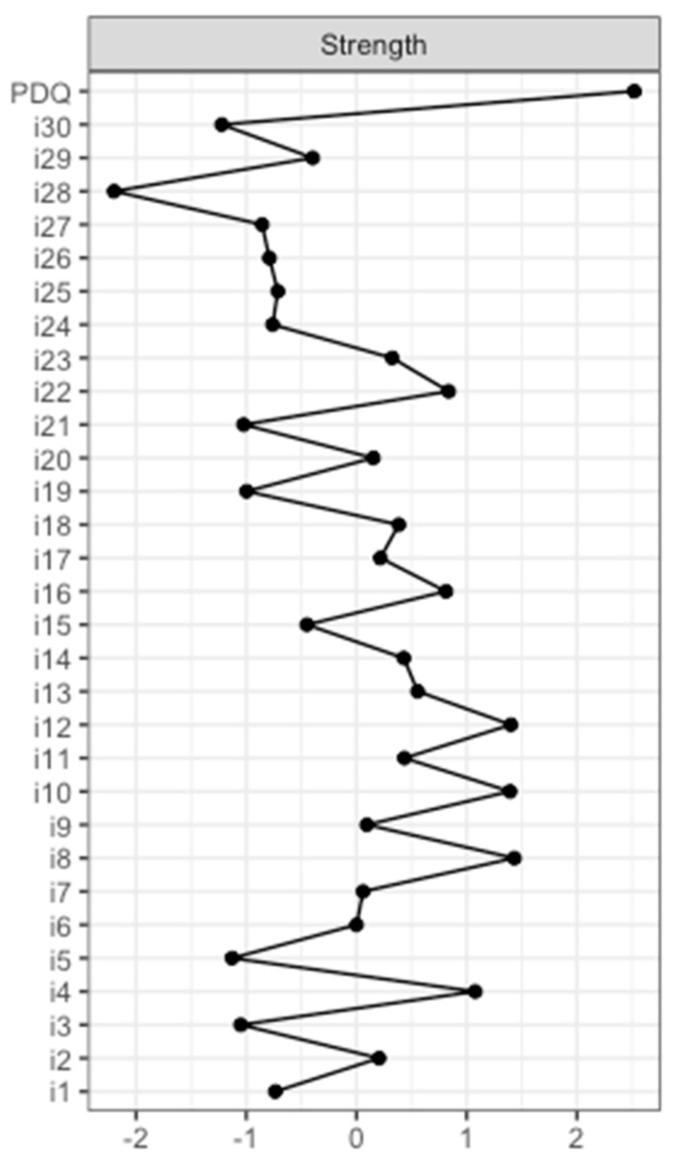
Node strength of the NMSS and PDQ-39 summary index. The strength centrality measures of PDQ-39 summary index (*PDQ*) and the items of the NMSS (*i1–i30*) are given in standardized values. Item 1: light headedness; item 2: fainting; item 3: daytime sleepiness; item 4: fatigue; item 5: sleep initiation; item 6: restless legs; item 7: loss of interest; item 8: lack of motivation; item 9: feeling nervous; item 10: feeling sad; item 11: flat mood; item 12: anhedonia; item 13: hallucinations; item 14: delusions; item 15: diplopia; item 16: concentration; item 17: forgetfulness; item 18: forget to do things; item 19: sialorrhea; item 20: dysphagia; item 21: constipation; item 22: urgency; item 23: frequency; item 24: nocturia; item 25: interest; item 26: problems having sex; item 27: pain; item 28: taste/smell; item 29: weight change; and item 30: hyperhidrosis. NMSS: Nonmotor Symptoms Scale in Parkinson´s disease. PDQ-39: Parkinson´s Disease Questionnaire 39.

In addition, the strength centrality measure of each node was determined. These values are shown in Figure 2 (and tabulated in Table S1). *PDQ* was determined to have the greatest strength. Accordingly, this node had the highest input weights from being directly connected other items. Respectively, the PDQ-39 summary index is of central importance to the complex interacting network of nonmotor symptoms of the NMSS. The nodewise predictability results revealed that 52.8% of the variance in the PDQ-39 summary index could be explained by the connected NMSS items (see Table S1). 

The case-dropping bootstrapped procedure revealed that the centrality measure strength remained high (CS (cor = 0.7) = 0.67; Figure S1), and accordingly, the network can be considered stable. 

#### 3.2.2. PDQ-39 Subscales

In addition to the aforementioned analysis of the PDQ-39 summary index, we conducted separate network analyses of the eight PDQ-39 subscales (MOB, ADL, EMO, STI, SOC, COG, COM, and BOD). The network structures between the nonmotor symptoms and each of the eight PDQ-39 subscales are shown in Figures S2–S9. The white node displays the PDQ-39 subscale (*MOB*, *ADL*, *EMO*, *STI*, *SOC*, COG, *COM*, or *BOD*), and the orange nodes display the items of the NMSS (*i1–i30).*

In general, all eight networks were found to be well-connected without isolated nodes. As revealed by the nodewise predictability analyses, the explained variances of the MOB, EMO, COG, and BOD subscales were high (R2: 38.5–52.7%) (see Table S2).

Moreover, strength centrality measures for each node within the eight networks were determined (Table S3). The COG subscale had the highest strength within its network and a high impact from directly connected nonmotor symptoms. We also examined the edge weights of each network (Table S3). It became apparent that different items of the NMSS are most highly associated with individual subscale measures. Within the “mobility” and “activities of daily living” network, the highest associations of the nodes *MOB* and *ADL* are with item 4 (*fatigue*) (edge weights 0.251 and 0.121). *Feeling sad* (item 10) is most highly associated with the node *EMO* (edge weights 0.276) in the “emotional well-being” network. Within the “stigma” network, the edge weight between *STI* and item 13 (*hallucinations*) is the highest (0.067), but it is still relatively low in comparison with the other subscales. *Loss of interest* (item 7) is highly connected to *SOC* (“social support” network) with an edge weight of 0.157. Regarding the “cognition” network, edge weight analyses two highly connected items were revealed: the edge weights between *COG* and both impaired *concentration* (item 16) and *daytime sleepiness* (item 3) were very high (edge weights 0.336 and 0.293, respectively). *Daytime sleepiness* (item 3) was also the item with the highest edge weight to *COM* in the “communication” network (edge weight 0.091). Finally, *hyperhidrosis* (item 30) seems to have the most significant influence on *BOD* in the “bodily discomfort” network with an edge weight of 0.220. 

Case-dropping bootstrapped procedures revealed that the centrality measure strength remained high for every subscale network (CS (cor = 0.7) > 0.5), and accordingly, the networks can be considered stable (see Table S2). 

#### 3.2.3. Bridge Strength

We identified nonmotor symptoms associated with the PDQ-39 summary index and particular subscales of the PDQ-39. However, it is particularly interesting to identify the nonmotor symptoms most associated with all eight PDQ-39 subscales. In this regard, the bridge strength of the NMSS items was determined. 

**Figure 3:**
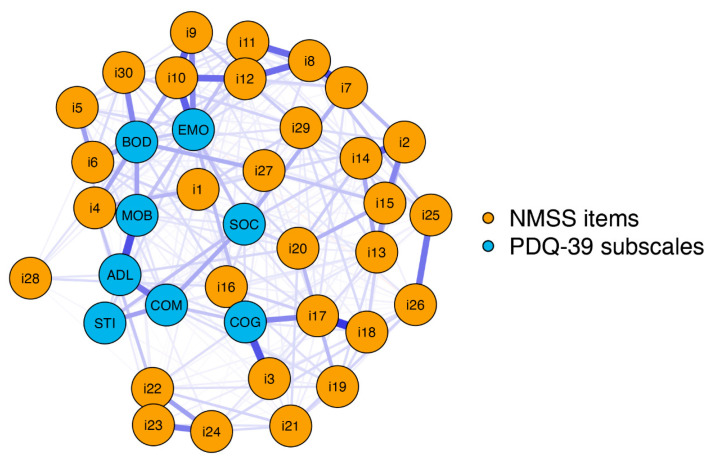
Network structure of the NMSS and PDQ-39 subscales. The blue nodes display the PDQ-39 subscales (*MOB*, *ADL*, *EMO*, *STI*, *SOC*, *COG*, *COM*, and *BOD*), and the orange nodes display the items included in the NMSS (*i1–i30*). The thickness of the edges indicates the strength of the correlations between these nodes. Item 1: light headedness; item 2: fainting; item 3: daytime sleepiness; item 4: fatigue; item 5: sleep initiation; item 6: restless legs; item 7: loss of interest; item 8: lack of motivation; item 9: feeling nervous; item 10: feeling sad; item 11: flat mood; item 12: anhedonia; item 13: hallucinations; item 14: delusions; item 15: diplopia; item 16: concentration; item 17: forgetfulness; item 18: forget to do things; item 19: sialorrhea; item 20: dysphagia; item 21: constipation; item 22: urgency; item 23: frequency; item 24: nocturia; item 25: interest; item 26: problems having sex; item 27: pain; item 28: taste/smell; item 29: weight change; and item 30: hyperhidrosis. PDQ-39 subscale coding: MOB, mobility; ADL, activities of daily living; EMO, emotional well-being; STI, stigma; SOC, social support; COG, cognition; COM, communication; BOD, bodily discomfort. NMSS: Nonmotor Symptoms Scale in Parkinson´s disease. PDQ-39: Parkinson´s Disease Questionnaire 39.

Therefore, we conducted a network analysis including the 30 NMSS items and eight PDQ-39 subscales (MOB, ADL, EMO, STI, SOC, COG, COM, BOD). The network structure is shown in Figure 3. The blue nodes display the PDQ-39 subscales (*MOB*, *ADL*, *EMO*, *STI*, SOC, *COG*, *COM*, or *BOD*), and the orange nodes display the NMSS items (*i1–i30*). 

**Figure 4:**
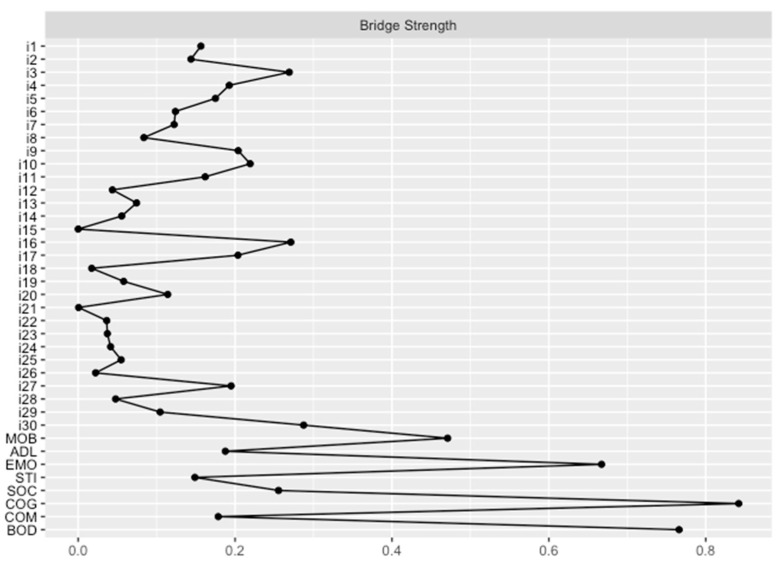
Bridge strengths of the NMSS and PDQ-39 subscales. The bridge strength indicates the sum of the values of all edges that exist between a node of one community and all nodes from another community. Bridge strength values are given for the nodes of the NMSS (*i1–i30*) and PDQ-39 subscales (*MOB*, *ADL*, *EMO*, *STI*, *SOC*, *COG*, *COM*, and *BOD*). Item 1: light headedness; item 2: fainting; item 3: daytime sleepiness; item 4: fatigue; item 5: sleep initiation; item 6: restless legs; item 7: loss of interest; item 8: lack of motivation; item 9: feeling nervous; item 10: feeling sad; item 11: flat mood; item 12: anhedonia; item 13: hallucinations; item 14: delusions; item 15: diplopia; item 16: concentration; item 17: forgetfulness; item 18: forget to do things; item 19: sialorrhea; item 20: dysphagia; item 21: constipation; item 22: urgency; item 23: frequency; item 24: nocturia; item 25: interest; item 26: problems having sex; item 27: pain; item 28: taste/smell; item 29: weight change; and item 30: hyperhidrosis. PDQ-39 subscale coding: MOB, mobility; ADL, activities of daily living; EMO, emotional well-being; STI, stigma; SOC, social support; COG, cognition; COM, communication; BOD, bodily discomfort. NMSS: Nonmotor Symptoms Scale in Parkinson´s disease. PDQ-39: Parkinson´s Disease Questionnaire 39.

The network analysis revealed a well-connected network (290 of 703 nonzero edges) without isolated nodes. The bridge strength centrality measures are shown in Figure 4. Accordingly, consideration of the community of NMSS items revealed that items 30 (*hyperhidrosis*), 16 (*concentration*), and 3 (*daytime sleepiness*) had the highest bridge strengths. These items are, in turn, associated with the subscales “bodily discomfort” (*hyperhidrosis*) and “cognition” (*concentration* and *daytime sleepiness*). 

The case-dropping bootstrapped procedure confirms that the network can be considered stable because the CS coefficient of the bridge strength remains high (CS (cor = 0.7) = 0.67) (see Figure S10). 

## 4. Discussion

Our study used network analyses to reveal the overall pattern of connections and, accordingly, the complex interactions between nonmotor symptoms and HRQoL in PD patients. We were able to demonstrate that the 30 items of the NMSS mainly influence the MOB, EMO, COG, and BOD subscales of the PDQ-39 due to the high predictability of these subscales. Moreover, considering the variety of other confounding nonmotor symptoms and HRQoL measures, we identified symptoms that were positively associated with overall HRQoL and symptoms that affect specific domains of HRQoL.

In summary, network analyses indicated that *fatigue*, *feeling sad*, *hyperhidrosis*, impaired *concentration*, and *daytime sleepiness* are the most influential nonmotor symptoms related to HRQoL. *Loss of interest* and *hallucinations* are also relevant, although their impacts are less pronounced because of the lower predictability of the STI and SOC subscales by the NMSS. 

The effect of fatigue on HRQoL can be interpreted in line with previous research. It is already known that fatigue is an early symptom of PD [37,38] that has a negative impact on quality of life [39,40,41]. Our study confirmed that *fatigue* is of central importance to the PDQ-39 summary index, and particularly for the MOB subscale. Accordingly, fatigue can be considered an influential nonmotor symptom. Therefore, identifying and subsequently treating fatigue in PD patients may be a promising way to improve HRQoL. However, evidence-based treatment strategies for fatigue remain limited [39]. As fatigue is correlated with depression in PD patients [41], treating any associated depressive symptoms may have a positive effect. 

Previous studies have revealed that depression is a strong determinant of a low HRQoL [42,43]. In addition, it was recently shown that depressive symptoms, particularly feelings of sadness, are of major importance within the complex network of nonmotor symptoms in PD [20]. However, until now, it has not been clarified whether depressive symptoms, even in the absence of depression, are also important for HRQoL, considering their complex interactions. In this study, we found that feeling sad had the greatest impact on the EMO subscale of the PDQ-39 and may represent a potential therapeutic target for improving HRQoL. Accordingly, close attention should be paid to depressive symptoms when treating patients with PD, especially because they are often heterogeneous and under-recognized [44,45]. 

Sweating disturbances are common in PD [46]. Axial hyperhidrosis may compensate for reduced sympathetic function in the extremities [47]. Our study demonstrates that *hyperhidrosis* primarily affects the BOD subscale of the PDQ-39 and has the highest bridge strength. Accordingly, hyperhidrosis was shown to have a meaningful impact on all eight subscales of the PDQ-39, which was not directly visible when looking at the network structure of the NMSS items and the PDQ-39 subscales (Figure 3). Nevertheless, this complex effect can be explained by the known relationship between hyperhidrosis and various other autonomic symptoms [48,49]. In general, our data suggest that the consideration of hyperhidrosis is beneficial, as it is a simple clinical screening tool to identify PD patients with autonomic symptoms [48] and could be used to uncover a possible therapeutic target to improve HRQoL. 

Excessive daytime sleepiness is a frequent problem in patients with PD [50]. Its pathophysiology is multifactorial and is caused by the degeneration of neurons controlling wakefulness, medications and their side effects, and even poor nocturnal sleep [51]. A previous study showed that PD patients with excessive daytime sleepiness had more severe nonmotor symptoms and a lower PDQ-39 summary index than those without excessive daytime sleepiness [52]. Moreover, excessive daytime sleepiness has been shown to be associated with cognitive impairment [51]. Our study strengthened this finding and showed that *daytime sleepiness* is strongly related to the COG subscale of the PDQ-39 and is a bridge symptom of HRQoL.

Furthermore, our study demonstrated that impaired *concentration* is strongly related to the COG subscale of the PDQ-39 and represents a bridge symptom of HRQoL. Although the high impact of *concentration* on the cognition domain of HRQoL is not surprising, our study revealed a broader influence of *concentration* on all eight subscales of the PDQ-39 and, thus, a multidimensional impact on HRQoL.

Our study provides new insights into the complex interacting network of nonmotor symptoms on HRQoL in PD patients. Nevertheless, our study has several limitations. First, the generated data are not fully representative of the PD population due to the inclusion and exclusion criteria used (i.e., age limit, no dementia, no severe comorbidities, and no second-line therapies) [22]. Accordingly, the results apply to the studied cohort only. However, the estimation of a stable network usually requires a larger sample size, which limits its applicability to smaller local cohorts. Second, nonmotor symptoms were recorded on a scale, and perceptions of nonmotor symptoms may have depended on the participants’ mood and motivation. In this regard, nonmotor fluctuations were not considered. Finally, network analysis remains an exploratory approach that cannot be used to determine causality. Thus, further research is needed to confirm whether influencing nonmotor symptoms with the highest impacts positively improve HRQoL. 

## 5. Conclusions

The network analysis revealed complex interactions between nonmotor symptoms and HRQoL in PD patients. *Fatigue*, *feeling sad*, *hyperhidrosis*, impaired *concentration*, and *daytime sleepiness* were shown to be the most influential nonmotor symptoms. Further research is needed to confirm whether influencing these nonmotor symptoms could improve HRQoL.

## Figures and Tables

**Table 1:** Descriptive statistics.

	Study Population
N	689
Sex	
Male	414 (60.1)
Female	275 (39.9)
Age	64 (57–70)
HY off	2 (2–2)
UPDRS III off	21 (14–30)
PDQ-39 summary index	12.8 (7.7–24.4)
Mobility, MOB	10.0 (2.5–25.0)
Activities of daily living, ADL	12.5 (4.2–25.0)
Emotional well-being, EMO	16.6 (4.2–33.3)
Stigma, STI	0.0 (0.0–25.0)
Social support, SOC	0.0 (0.0–8.3)
Cognition, COG	18.7 (6.3–31.2)
Communication, COM	0.0 (0.0–16.6)
Bodily discomfort, BOD	25.0 (8.3–41.6)
NMSS, total score	35 (19–61)
Cardiovascular (domain 1)	0 (0–2)
1. Light headedness	0 (0–2)
2. Fainting	0 (0–0)
Sleep/fatigue (domain 2)	6 (2–12)
3. Daytime sleepiness	1 (0–3)
4. Fatigue	1 (0–4)
5. Sleep initiation	0 (0–2)
6. Restless legs	0 (0–2)
Mood/apathy (domain 3)	3 (0–11)
7. Loss of interest	0 (0–1)
8. Lack of motivation	0 (0–1)
9. Feeling nervous	0 (0–2)
10. Feeling sad	0 (0–2)
11. Flat mood	0 (0–1)
12. Anhedonia	0 (0–2)
Perceptual (domain 4)	0 (0–1)
13. Hallucinations	0 (0–0)
14. Delusions	0 (0–0)
15. Diplopia	0 (0–0)
Attention/memory (domain 5)	2 (0–5)
16. Concentration	0 (0–2)
17. Forgetfulness	0 (0–2)
18. Forget to do things	0 (0–1)
Gastrointestinal (domain 6)	2 (0–5)
19. Sialorrhea	0 (0–1)
20. Dysphagia	0 (0–0)
21. Constipation	0 (0–2)
Urinary (domain 7)	6 (1.5–12)
22. Urgency	1 (0–6)
23. Frequency	1 (0–4)
24. Nocturia	1 (0–4)
Sexual dysfunction (domain 8)	1 (0–8)
25. Interest	0 (0–4)
26. Problems having sex	0 (0–4)
Miscellaneous (domain 9)	5 (1–12)
27. Pain	0 (0–1)
28. Taste/smell	2 (0–6)
29. Weight change	0 (0–0)
30. Hyperhidrosis	0 (0–1)

The number of participants is given in absolute values; categorical parameters are given as absolute values and percentages; other values are given as medians and interquartile ranges. HY, Hoehn and Yahr stage; N, number of participants; NMSS, Nonmotor Symptoms Scale in Parkinson’s disease; PDQ-39, Parkinson´s Disease Questionnaire 39; UPDRS, Unified Parkinson’s Disease Rating Scale.

## Data Availability

The data presented in this study are available on request from D.S.-G. on behalf of the COPPADIS Study Group.

## Supplementary Materials

The following supporting information can be downloaded at: https://www.mdpi.com/article/10.3390/jcm12072573/s1, Figure S1: Case-dropping bootstrap strength of the Nonmotor Symptoms Scale in Parkinson’s disease (NMSS) and Parkinson´s Disease Questionnaire 39 (PDQ-39) summary index; Figure S2: Network structure of the NMSS and MOB; Figure S3: Network structure of the NMSS and ADL; Figure S4: Network structure of the NMSS and EMO; Figure S5: Network structure of the NMSS and STI; Figure S6: Network structure of the NMSS and SOC; Figure S7: Network structure of the NMSS and COG; Figure S8: Network structure of the NMSS and COM; Figure S9: Network structure of the NMSS and BOD; Figure S10: Case-dropping bootstrap bridge strength of the NMSS and PDQ-39 subscales; Table S1: Network analysis of the PDQ-39 summary index; Table S2: Network characteristics of the PDQ-39 subscales; Table S3: Network analyses of the PDQ-39 subscales. Table S4: Affiliations of the collaborators of the COPPADIS Study Group.

## Appendix A

The Collaborators of the COPPADIS Study Group (affiliations are shown in Table S4): Adarmes, A.D.; Almeria, M.; Alonso Losada, M.G.; Alonso Cánovas, A.; Alonso Frech, F.; Alonso Redondo, R.; Álvarez, I.; Álvarez Sauco, M.; Aneiros Díaz, A.; Arnáiz, S.; Arribas, S.; Ascunce Vidondo, A.; Aguilar, M.; Ávila, M.A.; Bernardo Lambrich, N.; Bejr-Kasem, H.; Blázquez Estrada, M.; Botí, M.; Borrue, C.; Buongiorno, M.T.; Cabello González, C.; Cabo López, I.; Caballol, N.; Cámara Lorenzo, A.; Canfield Medina, H.; Carrillo, F.; Carrillo Padilla, F.J.; Casas, E.; Catalán, M.J.; Clavero, P.; Cortina Fernández, A.; Cosgaya, M.; Cots Foraster, A.; Crespo Cuevas, A.; Cubo, E.; de Deus Fonticoba, T.; de Fábregues-Boixar, O.; Díez-Fairen, M.; Dotor García-Soto, J.; Erro, E.; Escalante, S.; Estelrich Peyret, E.; Fernández Guillán, N.; Gámez, P.; Gallego, M.; García Caldentey, J.; García Campos, C.; García Díez, C.; García Moreno, J.M.; Gastón, I.; Gómez Garre, M.P.; Gómez Mayordomo, V.; González Aloy, J.; González-Aramburu, I.; González Ardura, J.; González García, B.; González Palmás, M.J.; González Toledo, G.R.; Golpe Díaz, A.; Grau Solá, M.; Guardia, G.; Hernández Vara, J.; Horta-Barba, A.; Idoate Calderón, D.; Infante, J.; Jesús, S.; Kulisevsky, J.; Kurtis, M.; Labandeira, C.; Labrador, M.A.; Lacruz, F.; Lage Castro, M.; Lastres Gómez, S.; Legarda, I.; López Ariztegui, N.; López Díaz, L.M.; López Domínguez, D.; López Manzanares, L.; López Seoane, B.; Lucas del Pozo, S.; Macías, Y.; Mata, M.; Martí Andres, G.; Martí, M.J.; Martínez Castrillo, J.C.; Martinez-Martin, P.; McAfee, D.; Meitín, M.T.; Mendoza Plasencia, Z.; Menéndez González, M.; Méndez del Barrio, C.; Mir, P.; Miranda Santiago, J.; Morales Casado, M.I.; Moreno Diéguez, A.; Nogueira, V.; Novo Amado, A.; Novo Ponte, S.; Ordás, C.; Pagonabarraga, J.; Pareés, I.; Pascual-Sedano, B.; Pastor, P.; Pérez Fuertes, A.; Pérez Noguera, R.; Planas-Ballvé, A.; Planellas, L.; Prats, M.A.; Prieto Jurczynska, C.; Puente, V.; Pueyo Morlans, M.; Puig Daví, A.; Redondo Rafales, N.; Rodríguez Méndez, L.; Rodríguez Pérez, A.B.; Roldán, F.; Ruíz De Arcos, M.; Ruíz Martínez, J.; Sánchez Alonso, P.; Sánchez-Carpintero, M.; Sánchez Díez, G.; Sánchez Rodríguez, A.; Santacruz, P.; Santos García, D.; Segundo Rodríguez, J.C.; Seijo, M.; Sierra Peña, M.; Solano Vila, B.; Suárez Castro, E.; Tartari, J.P.; Valero, C.; Vargas, L.; Vela, L.; Villanueva, C.; Vives, B.
