# Supplementary material for: The Impact of Nonmotor Symptoms on Health-Related Quality of Life in Parkinson’s Disease: A Network Analysis Approach
Source: J Clin Med. 2023 Mar 29;12(7):2573. doi: 10.3390/jcm12072573 (PMC10094983; doi:10.3390/jcm12072573)
Supplement: Supplementary file 1 [file jcm-12-02573-s001.zip › jcm-2255110-supplementary.pdf]

Supplementary Materials

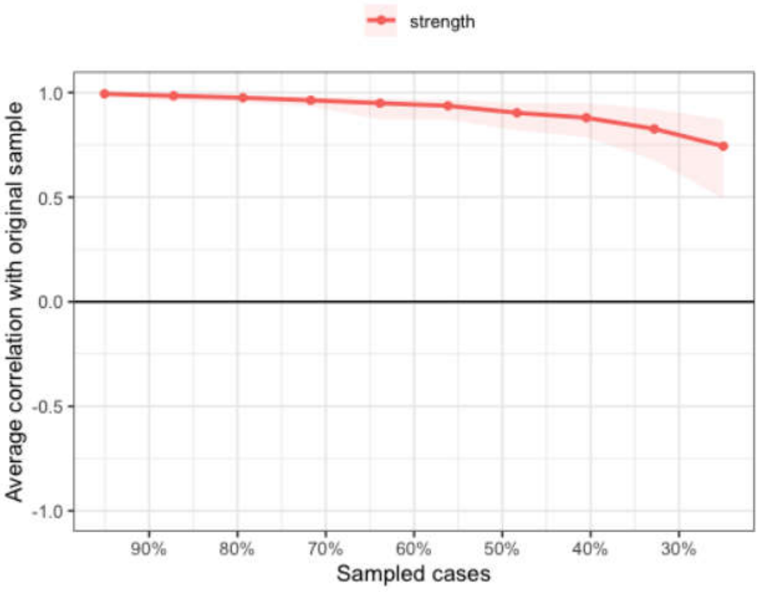

Figure S1. Case-dropping bootstrap strength of the Nonmotor Symptoms Scale in Parkinson's disease (NMSS) and Parkinson's Disease Questionnaire 39 (PDQ-39) summary index (number of bootstraps = 1000). Correlations of the strength centrality measure between the original sample and those of the subsamples with increasingly higher percentages of dropout cases were calculated. The correlation stability coefficient (CS coefficient) represents the maximum proportion of cases that can be dropped to retain a correlation of at least 0.70 with the original strength in at least 95% of the samples. The 95% confidence interval of the correlation is indicated. The case-dropping bootstrap procedure showed that the CS coefficient of the node strength (CS (cor = 0.7) = 0.67) remained stable.

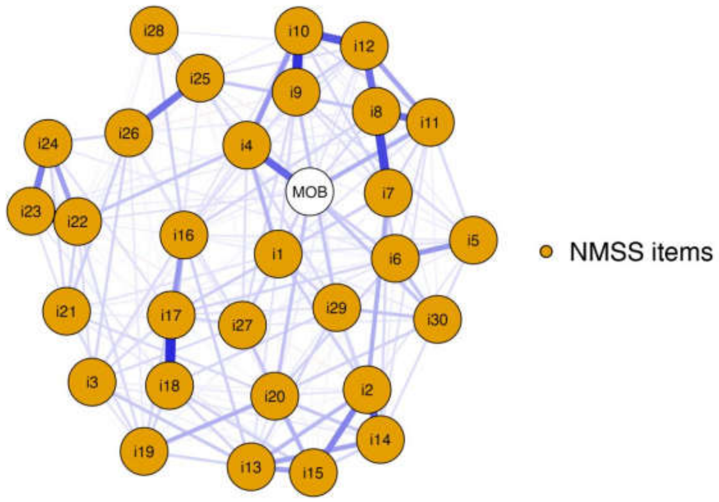

Figure S2: Network structure of the NMSS and MOB. The node *MOB* displays the PDQ-39 mobility subscale, and nodes *i1–i30* display the items included in the NMSS (orange). The thickness of the edges indicates the strengths of the correlations between these nodes. Item 1: light headedness; item 2: fainting; item 3: daytime sleepiness; item 4: fatigue; item 5: sleep initiation; item 6: restless legs; item 7: loss of interest; item 8: lack of motivation; item 9: feeling nervous; item 10: feeling sad; item 11: flat mood; item 12: anhedonia; item 13: hallucinations; item 14: delusions; item 15: diplopia; item 16: concentration; item 17: forgetfulness; item 18: forget to do things; item 19: sialorrhea; item 20: dysphagia; item 21: constipation; item 22: urgency; item 23: frequency; item 24: nocturia; item 25: interest; item 26: problems having sex; item 27: pain; item 28: taste/smell; item 29: weight change;

and item 30: hyperhidrosis. NMSS: Nonmotor Symptoms Scale in Parkinson´s disease. PDQ-39: Parkinson´s Disease Questionnaire 39. MOB: mobility subscale.

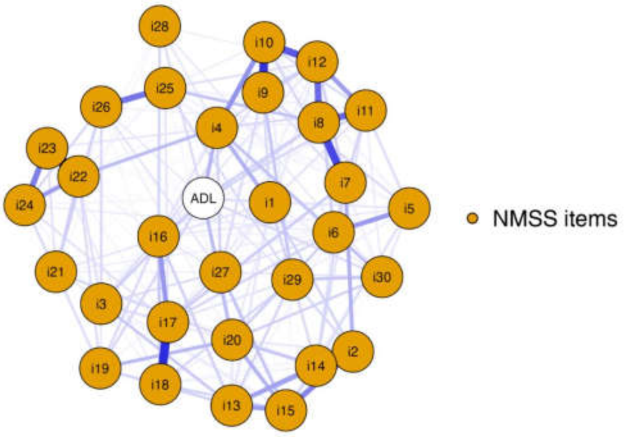

Figure S3: Network structure of the NMSS and ADL. The node *ADL* displays the PDQ-39 activities of daily living subscale, and nodes *i1–i30* display the items included in the NMSS (orange). The thickness of the edges indicates the strengths of the correlations between these nodes. Item 1: light headedness; item 2: fainting; item 3: daytime sleepiness; item 4: fatigue; item 5: sleep initiation; item 6: restless legs; item 7: loss of interest; item 8: lack of motivation; item 9: feeling nervous; item 10: feeling sad; item 11: flat mood; item 12: anhedonia; item 13: hallucinations; item 14: delusions; item 15: diplopia; item 16: concentration; item 17: forgetfulness; item 18: forget to do things; item 19: sialorrhea; item 20: dysphagia; item 21: constipation; item 22: urgency; item 23: frequency; item 24: nocturia; item 25: interest; item 26: problems having sex; item 27: pain; item 28: taste/smell; item 29: weight change; and item 30: hyperhidrosis. NMSS: Nonmotor Symptoms Scale in Parkinson´s disease. PDQ-39: Parkinson´s Disease Questionnaire 39. ADL: activities of daily living subscale.

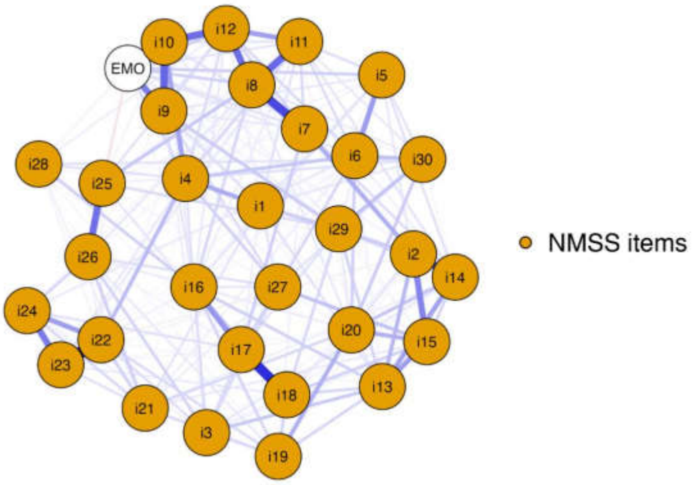

Figure S4: Network structure of the NMSS and EMO. The node *EMO* displays the PDQ-39 emotional well-being subscale, and nodes *i1–i30* display the items included in the NMSS (orange). The thickness of the edges indicates the strengths of the correlations between these nodes. Item 1: light headedness; item 2: fainting; item 3: daytime sleepiness; item 4: fatigue; item 5: sleep initiation; item 6: restless legs; item 7: loss of interest; item 8: lack of motivation; item 9: feeling nervous; item 10: feeling sad; item 11: flat mood; item 12: anhedonia; item 13: hallucinations; item 14: delusions; item 15: diplopia; item 16: concentration; item 17: forgetfulness; item 18: forget to do things; item 19: sialorrhea; item 20: dysphagia; item 21: constipation; item 22: urgency; item 23: frequency; item 24: nocturia; item 25: interest; item 26: problems having sex; item 27: pain; item 28: taste/smell; item 29:

weight change; and item 30: hyperhidrosis. NMSS: Nonmotor Symptoms Scale in Parkinson´s disease. PDQ-39: Parkinson´s Disease Questionnaire 39. EMO: emotional well-being subscale.

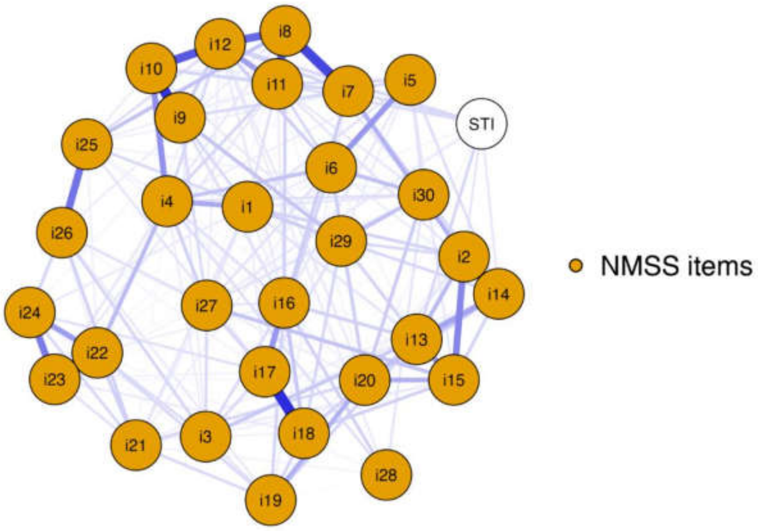

Figure S5: Network structure of the NMSS and STI. The node *STI* displays the PDQ-39 stigma subscale, and nodes *i1–i30* display the items included in the NMSS (orange). The thickness of the edges indicates the strengths of the correlations between these nodes. Item 1: light headedness; item 2: fainting; item 3: daytime sleepiness; item 4: fatigue; item 5: sleep initiation; item 6: restless legs; item 7: loss of interest; item 8: lack of motivation; item 9: feeling nervous; item 10: feeling sad; item 11: flat mood; item 12: anhedonia; item 13: hallucinations; item 14: delusions; item 15: diplopia; item 16: concentration; item 17: forgetfulness; item 18: forget to do things; item 19: sialorrhea; item 20: dysphagia; item 21: constipation; item 22: urgency; item 23: frequency; item 24: nocturia; item 25: interest; item 26: problems having sex; item 27: pain; item 28: taste/smell; item 29: weight change; and item 30: hyperhidrosis. NMSS: Nonmotor Symptoms Scale in Parkinson´s disease. PDQ-39: Parkinson´s Disease Questionnaire 39. STI: stigma subscale.

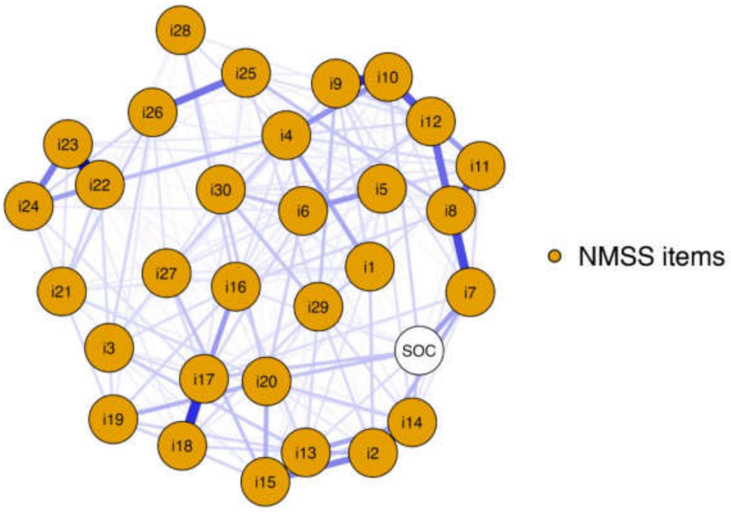

Figure S6: Network structure of the NMSS and SOC. The node *SOC* displays the PDQ-39 social support subscale, and nodes *i1–i30* display the items included in the NMSS (orange). The thickness of the edges indicates the strengths of the correlations between these nodes. Item 1: light headedness; item 2: fainting; item 3: daytime sleepiness; item 4: fatigue; item 5: sleep initiation; item 6: restless legs; item 7: loss of interest; item 8: lack of motivation; item 9: feeling nervous; item 10: feeling sad; item 11: flat mood; item 12: anhedonia; item 13: hallucinations; item 14: delusions; item

15: diplopia; item 16: concentration; item 17: forgetfulness; item 18: forget to do things; item 19: sialorrhea; item 20: dysphagia; item 21: constipation; item 22: urgency; item 23: frequency; item 24: nocturia; item 25: interest; item 26: problems having sex; item 27: pain; item 28: taste/smell; item 29: weight change; and item 30: hyperhidrosis. NMSS: Nonmotor Symptoms Scale in Parkinson´s disease. PDQ-39: Parkinson´s Disease Questionnaire 39. SOC: social support subscale.

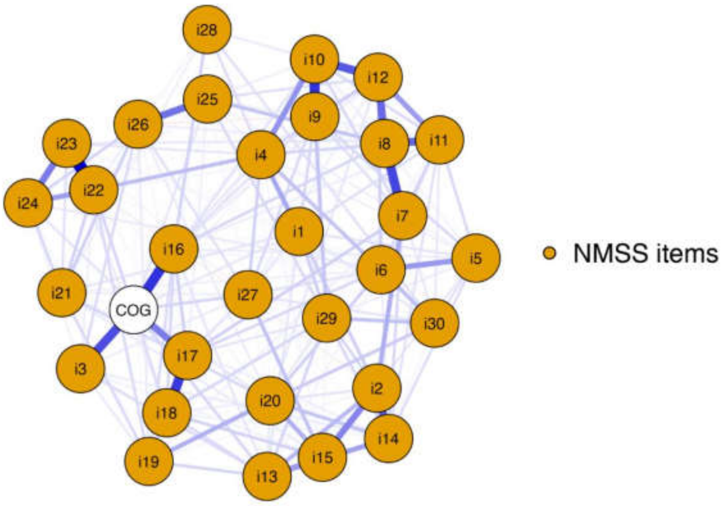

Figure S7: Network structure of the NMSS and COG. The node *COG* displays the PDQ-39 cognition subscale, and nodes *i1–i30* display the items included in the NMSS (orange). The thickness of the edges indicates the strengths of the correlations between these nodes. Item 1: light headedness; item 2: fainting; item 3: daytime sleepiness; item 4: fatigue; item 5: sleep initiation; item 6: restless legs; item 7: loss of interest; item 8: lack of motivation; item 9: feeling nervous; item 10: feeling sad; item 11: flat mood; item 12: anhedonia; item 13: hallucinations; item 14: delusions; item 15: diplopia; item 16: concentration; item 17: forgetfulness; item 18: forget to do things; item 19: sialorrhea; item 20: dysphagia; item 21: constipation; item 22: urgency; item 23: frequency; item 24: nocturia; item 25: interest; item 26: problems having sex; item 27: pain; item 28: taste/smell; item 29: weight change; and item 30: hyperhidrosis. NMSS: Nonmotor Symptoms Scale in Parkinson´s disease. PDQ-39: Parkinson´s Disease Questionnaire 39. COG: cognition subscale.

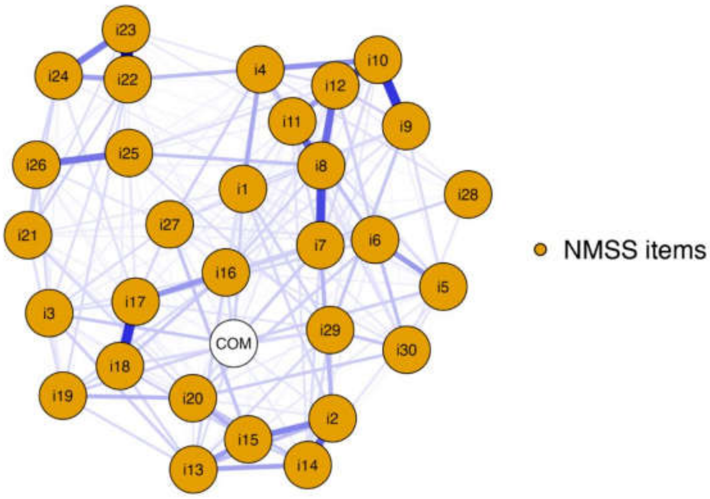

Figure S8: Network structure of the NMSS and COM. The node *COM* displays the PDQ-39 communication subscale, and nodes *i1–i30* display the items included in the NMSS (orange). The thickness of the edges indicates the strengths of the correlations between these nodes. Item 1: light headedness; item 2: fainting; item 3: daytime sleepiness; item 4: fatigue; item 5: sleep initiation; item 6:

restless legs; item 7: loss of interest; item 8: lack of motivation; item 9: feeling nervous; item 10: feeling sad; item 11: flat mood; item 12: anhedonia; item 13: hallucinations; item 14: delusions; item 15: diplopia; item 16: concentration; item 17: forgetfulness; item 18: forget to do things; item 19: sialorrhea; item 20: dysphagia; item 21: constipation; item 22: urgency; item 23: frequency; item 24: nocturia; item 25: interest; item 26: problems having sex; item 27: pain; item 28: taste/smell; item 29: weight change; and item 30: hyperhidrosis. NMSS: Nonmotor Symptoms Scale in Parkinson´s disease. PDQ-39: Parkinson´s Disease Questionnaire 39. COM: communication subscale.

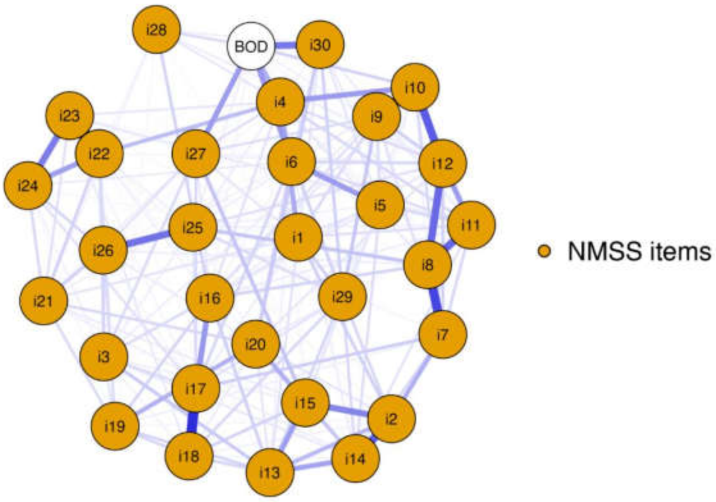

Figure S9: Network structure of the NMSS and BOD. The node *BOD* displays the PDQ-39 bodily discomfort subscale, and nodes *i1–i30* display the items included in the NMSS (orange). The thickness of the edges indicates the strengths of the correlations between these nodes. Item 1: light headedness; item 2: fainting; item 3: daytime sleepiness; item 4: fatigue; item 5: sleep initiation; item 6: restless legs; item 7: loss of interest; item 8: lack of motivation; item 9: feeling nervous; item 10: feeling sad; item 11: flat mood; item 12: anhedonia; item 13: hallucinations; item 14: delusions; item 15: diplopia; item 16: concentration; item 17: forgetfulness; item 18: forget to do things; item 19: sialorrhea; item 20: dysphagia; item 21: constipation; item 22: urgency; item 23: frequency; item 24: nocturia; item 25: interest; item 26: problems having sex; item 27: pain; item 28: taste/smell; item 29: weight change; and item 30: hyperhidrosis. NMSS: Nonmotor Symptoms Scale in Parkinson´s disease. PDQ-39: Parkinson´s Disease Questionnaire 39. BOD: bodily discomfort subscale.

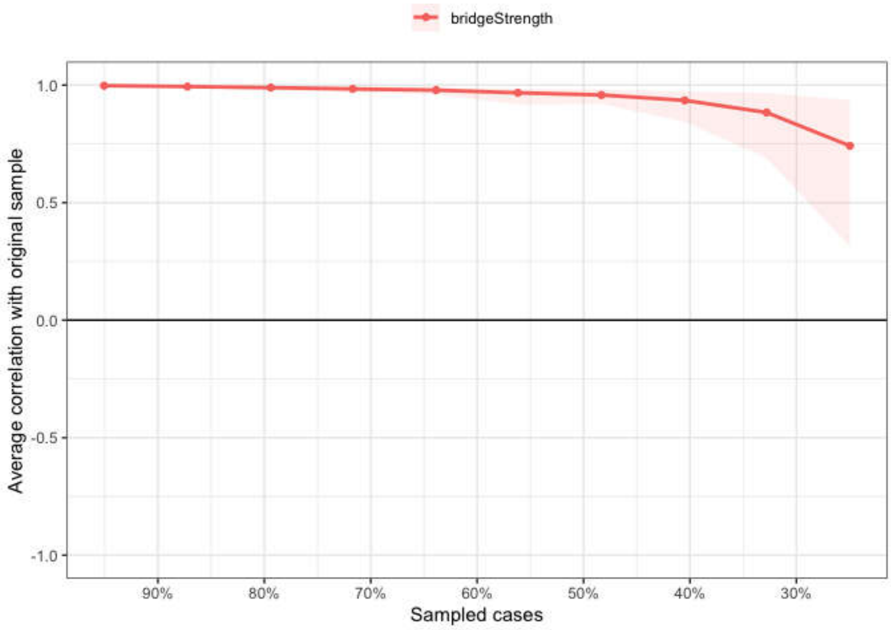

Figure S10: Case-dropping bootstrap bridge strength of the NMSS and PDQ-39 subscales (number of bootstraps = 1000). The correlations of the bridge strength centrality measure between the original sample and the subsamples with increasingly higher percentages of dropout cases were calculated. The correlation stability coefficient (CS coefficient) represents the maximum proportion of cases that can be dropped to retain a correlation of at least 0.70 with the original bridge strength in at least 95% of the samples. The 95% confidence interval of the correlation is indicated. The case-dropping bootstrap procedure showed that the CS coefficient of bridge strength (CS (cor = 0.7) = 0.67) remained stable. NMSS: Nonmotor Symptoms Scale in Parkinson´s disease. PDQ-39: Parkinson´s Disease Questionnaire 39.

Table S1: Network analysis of the PDQ-39 summary index.

Node	Strength	Predictability	Edge weight
PDQ	2.996	0.528	/
i1	−0.567	0.262	0.067
i2	0.662	0.411	0.000
i3	−1.072	0.185	0.086
i4	0.750	0.470	0.184
i5	−0.962	0.204	0.059
i6	−0.009	0.287	0.066
i7	0.151	0.477	0.007
i8	1.193	0.608	0.066
i9	0.082	0.427	0.040
i10	0.965	0.586	0.096
i11	0.577	0.496	0.099
i12	1.272	0.624	0.063
i13	0.312	0.401	0.025
i14	0.472	0.398	0.000
i15	0.103	0.285	0.000
i16	0.397	0.394	0.119
i17	0.664	0.370	0.037
i18	0.002	0.395	0.000
i19	−1.069	0.197	0.006
i20	0.154	0.282	0.106
i21	−1.121	0.185	0.010

i22	0.737	0.467	0.048
i23	0.067	0.448	0.011
i24	−0.857	0.272	0.000
i25	−0.738	0.247	0.023
i26	−0.766	0.197	0.000
i27	−0.714	0.205	0.107
i28	−2.440	0.087	0.072
i29	−0.236	0.279	0.042
i30	−1.004	0.185	0.092

The strength centrality measures and predictability are given nodewise for the PDQ-39 summary index (*PDQ*) and the items of the NMSS (*i1–i30*). The edge weights refer to the associations between the PDQ-39 summary index (*PDQ*) and each item of the NMSS (*i1–i30*). Item 1: light headedness; item 2: fainting; item 3: daytime sleepiness; item 4: fatigue; item 5: sleep initiation; item 6: restless legs; item 7: loss of interest; item 8: lack of motivation; item 9: feeling nervous; item 10: feeling sad; item 11: flat mood; item 12: anhedonia; item 13: hallucinations; item 14: delusions; item 15: diplopia; item 16: concentration; item 17: forgetfulness; item 18: forget to do things; item 19: sialorrhea; item 20: dysphagia; item 21: constipation; item 22: urgency; item 23: frequency; item 24: nocturia; item 25: interest; item 26: problems having sex; item 27: pain; item 28: taste/smell; item 29: weight change; and item 30: hyperhidrosis. NMSS: Nonmotor Symptoms Scale in Parkinson´s disease. PDQ-39: Parkinson´s Disease Questionnaire 39.

Table S2: Network characteristics of the PDQ-39 subscales.

Network PDQ-39 subscale	Nonzero edges	Predictability subscale	CS strength
MOB	231 of 465	0.385	0.672
ADL	228 of 465	0.252	0.595
EMO	233 of 465	0.527	0.672
STI	226 of 465	0.143	0.672
SOC	234 of 465	0.239	0.595
COG	229 of 465	0.501	0.672
COM	229 of 465	0.222	0.672
BOD	228 of 465	0.403	0.595

For every network in the eight subscales, the number of nonzero edges, predictability of each node representing the PDQ-39 subscale, and correlation stability coefficient (CS coefficient) of the strength centrality measure are given separately (number of bootstraps = 1000). The CS coefficient represents the maximum proportion of cases that can be dropped to retain a correlation of at least 0.70 with the original strength in at least 95% of the samples. CS coefficients > 0.5 indicate that the networks are stable. PDQ-39 subscale coding: MOB, mobility; ADL, activities of daily living; EMO, emotional well-being; STI, stigma; SOC, social support; COG, cognition; COM, communication; BOD, bodily discomfort. PDQ-39: Parkinson´s Disease Questionnaire 39.

Table S3: Network analyses of the PDQ-39 subscales.

	**MOB**		**ADL**		**EMO**		**STI**		SOC		COG		COM		BOD	
PDQ-39 subscale	1.052	/	0.316	/	1.476	/	-1.885	/	-0.497	/	2.276	/	0.251	/	0.732	/
i1	–0.552	0.050	–0.427	0.057	–0.564	0.030	–0.356	0.017	–0.486	0.018	–0.522	0.040	–0.402	0.078	–0.549	0.000
i2	0.758	0.000	0.352	0.000	0.760	0.000	0.528	0.040	0.689	0.017	0.714	0.000	0.401	0.039	0.661	–0.009
i3	–1.120	0.022	–1.014	0.078	–1.136	0.000	–0.954	0.000	–1.094	0.005	–1.167	0.293	–0.995	0.091	–1.141	0.000
i4	1.366	0.251	1.347	0.121	1.269	0.061	1.177	0.000	1.294	0.000	1.138	0.021	1.227	0.000	1.278	0.126
i5	–1.041	0.000	–0.944	0.035	–1.086	0.080	–0.850	0.000	–1.005	0.000	–0.964	0.012	–0.855	0.080	–1.064	0.013
i6	0.092	0.051	0.162	0.000	0.087	0.040	0.209	0.000	0.132	0.000	0.097	0.003	0.161	0.000	0.262	0.169
i7	0.154	0.000	0.209	0.000	0.112	0.051	0.432	0.060	0.497	0.157	0.143	0.000	0.394	0.083	0.182	0.000
i8	1.421	0.062	1.603	0.071	1.421	0.076	1.386	0.000	1.411	0.007	1.309	0.003	1.459	0.000	1.436	0.000
i9	0.216	0.000	0.090	0.003	0.130	0.210	0.155	0.000	0.192	0.000	0.150	0.000	0.071	0.000	0.159	0.005
i10	1.174	0.009	1.285	0.000	1.209	0.276	1.220	0.000	1.356	0.075	1.132	0.046	1.281	0.000	1.328	0.075
i11	0.835	0.133	0.998	0.069	0.740	0.044	1.042	0.056	0.860	0.029	0.763	0.000	0.906	0.008	0.846	0.011
i12	1.589	0.043	1.693	0.000	1.499	0.081	1.809	0.064	1.622	0.000	1.508	0.000	1.797	0.037	1.642	0.023
i13	0.369	0.000	0.404	0.000	0.354	0.000	0.630	0.067	0.467	0.056	0.216	0.067	0.460	0.056	0.386	0.000
i14	0.520	0.000	0.568	0.000	0.493	0.024	0.677	0.039	0.668	0.092	0.478	0.000	0.569	0.032	0.547	0.000
i15	0.094	0.013	0.142	0.011	0.083	0.000	0.173	0.000	0.114	0.000	0.084	0.000	0.112	0.000	0.105	0.000
i16	0.699	0.018	0.783	0.028	0.653	0.015	0.764	0.000	0.813	0.050	0.490	0.336	0.847	0.069	0.739	0.020
i17	0.846	0.000	0.876	0.000	0.827	0.000	0.886	0.007	0.996	0.091	0.520	0.216	0.873	0.009	0.852	0.000
i18	–0.033	0.000	0.004	0.000	–0.048	0.007	0.062	0.000	–0.033	0.000	–0.167	0.056	0.044	0.033	–0.027	0.000
i19	–1.229	0.000	–1.177	0.067	–1.233	0.000	–1.072	0.000	–1.213	0.000	–1.195	0.010	–1.158	0.078	–1.255	0.000
i20	0.344	0.100	0.492	0.110	0.309	0.014	0.446	0.017	0.507	0.099	0.264	0.039	0.419	0.055	0.333	0.000
i21	–1.283	0.000	–1.331	0.000	–1.283	0.000	–1.121	0.000	–1.247	0.003	–1.185	0.009	–1.287	0.024	–1.315	0.000
i22	0.924	0.045	0.727	0.000	0.906	0.000	0.781	0.000	0.945	0.016	0.795	0.069	0.751	0.000	0.873	0.000
i23	0.062	0.028	0.118	0.008	0.070	0.000	0.178	0.006	0.101	0.000	0.058	0.000	0.121	0.005	0.089	0.000
i24	–1.017	0.000	–0.975	0.016	–1.030	0.000	–0.847	0.000	–0.989	0.000	–0.957	0.000	–0.999	0.000	–1.032	0.000
i25	–0.839	0.000	–0.819	0.000	–0.839	0.040	–0.671	0.000	–0.807	0.000	–0.783	0.043	–0.815	0.000	–0.817	0.028
i26	–0.837	0.030	–1.058	0.035	–0.540	–0.041	–0.914	0.000	–0.860	0.000	–0.827	0.009	–1.063	0.026	–0.960	0.000
i27	–0.632	0.081	–0.587	0.028	–0.650	0.008	–0.389	0.036	–0.593	0.008	–0.701	0.068	–0.592	0.027	–0.504	0.162
i28	–2.744	0.023	–2.820	0.059	–2.726	0.019	–2.584	0.000	–2.742	0.000	–2.582	0.005	–2.920	0.000	–2.785	0.061
i29	–0.220	0.011	–0.111	0.040	–0.277	0.024	–0.102	0.000	–0.159	0.030	–0.208	0.000	–0.097	0.054	–0.179	0.075
i30	–0.969	0.097	–0.906	0.061	–0.985	0.017	–0.809	0.000	–0.936	0.000	–0.880	0.000	–0.961	0.000	–0.824	0.220

Strength centrality measures of the eight network analyses of the PDQ-39 subscales are given node-wise for the subscale variable (*MOB*, *ADL*, *EMO*, *STI*, *SOC*, *COG*, *COM*, and *BOD*) and the items of the NMSS (*i1*–*i30*). Edge weights refer to the associations between the PDQ-39 subscale variable and each of the items of the NMSS (*i1*–*i30*). Item 1: light headedness; item 2: fainting; item 3: daytime sleepiness; item 4: fatigue; item 5: sleep initiation; item 6: restless legs; item 7: loss of interest; item 8: lack of motivation; item 9: feeling nervous; item 10: feeling sad; item 11: flat mood; item 12: anhedonia; item 13: hallucinations; item 14: delusions; item 15: diplopia; item 16: concentration; item 17: forgetfulness; item 18: forget to do things; item 19: sialorrhea; item 20: dysphagia; item 21: constipation; item 22: urgency; item 23: frequency; item 24: nocturia; item 25: interest; item 26: problems having sex; item 27: pain; item 28: taste/smell; item 29: weight change; and item 30: hyperhidrosis. MOB, mobility; ADL, activities of daily living; EMO, emotional well-being; STI, stigma; SOC, social support; COG, cognition; COM, communication; BOD, bodily discomfort. PDQ-39: Parkinson´s Disease Questionnaire 39. NMSS: Non-Motor Symptoms Scale in Parkinson´s disease.

Table S4. Affiliations of the collaborators of the COPPADIS Study Group.

Name (Last Name, First Name)	Location	Role	Contribution
Astrid Adarmes, Daniela	Hospital Universitario Virgen del Rocío, Sevilla, Spain	Site investigator	Evaluation of participants and/or data management
Almeria, Marta	Hospital Universitari Mutua de Terrassa, Terrassa, Barcelona, Spain	Site investigator	Neuropsychologist; evaluation of participants
Alonso Losada, Maria Gema	Hospital Álvaro Cunqueiro, Complejo Hospitalario Universitario de Vigo (CHUVI), Vigo, Spain	Site investigator/PI	Coordination at the center Evaluation of participants and/or data management
Alonso Cánovas, Araceli	Hospital Universitario Ramón y Cajal, Madrid, Spain	Site investigator	Evaluation of participants and/or data management
Alonso Frech, Fernando	Hospital Universitario Clínico San Carlos, Madrid, Spain	Site investigator	Evaluation of participants and/or data management
Alonso Redondo, Ruben	Hospital Universitario Lucus Augusti (HULA), Lugo, Spain	Site investigator/PI	Coordination at the center Evaluation of participants and/or data management
Aneiros Díaz, Ángel	Complejo Hospitalario Universitario de Ferrol (CHUF), Ferrol, A Coruña, Spain	Site investigator/PI	Coordination at the center Evaluation of participants and/or data management
Álvarez, Ignacio	Hospital Universitari Mutua de Terrassa, Terrassa, Barcelona, Spain	Site investigator	Evaluation of participants and/or data management
Álvarez Sauco, María	Hospital General Universitario de Elche, Elche, Spain	Site investigator/PI	Coordination at the center Evaluation of participants and/or data management
Arnáiz, Sandra	Complejo Asistencial Universitario de Burgos, Burgos, Spain	Site investigator	Evaluation of participants and/or data management
Arribas, Sonia	Hospital Universitari Mutua de Terrassa, Terrassa, Barcelona, Spain	Site investigator	Neuropsychologist; evaluation of participants
Ascunce Vidondo, Arancha	Complejo Hospitalario de Navarra, Pamplona, Spain	Site investigator	Evaluation of participants and/or data management
Aguilar, Miquel	Hospital Universitari Mutua de Terrassa, Terrassa, Barcelona, Spain	Site investigator	Evaluation of participants and/or data management

Ávila Rivera, Maria Asunción	Consorci Sanitari Integral, Hospital General de<br>L´Hospitalet, L´Hospitalet de Llobregat, Barcelona, Spain	Site investigator/PI	Coordination at the center<br>Evaluation of participants and/or data<br>management
Bernardo Lambrich, Noemí	Hospital de Tortosa Verge de la Cinta (HTVC),<br>Tortosa, Tarragona, Spain	Site investigator	Evaluation of participants and/or data<br>management
Bejr-Kasem, Helena	Hospital de Sant Pau, Barcelona, Spain	Site investigator	Evaluation of participants and/or data<br>management
Blázquez Estrada, Marta	Hospital Universitario Central de Asturias, Oviedo, Spain	Site investigator	Evaluation of participants and/or data<br>management
Botí González, Maria Ángeles	Hospital Universitari Mutua de Terrassa, Terrassa, Barcelona, Spain	Site investigator	Neuropsychologist; evaluation of participants
Borrué, Carmen	Hospital Infanta Sofía, Madrid, Spain	Site investigator/PI	Coordination at the center<br>Evaluation of participants and/or data<br>management
Buongiorno, Maria Teresa	Hospital Universitari Mutua de Terrassa, Terrassa, Barcelona, Spain	Site investigator	Nurse study coordinator
Cabello González, Carolina	Complejo Hospitalario de Navarra, Pamplona,<br>Spain	Site investigator	Scheduling of evaluations
Cabo López, Iria	Complejo Hospitalario Universitario de Pontevedra (CHOP), Pontevedra, Spain	Site investigator/PI	Coordination at the center<br>Evaluation of participants and/or data<br>management
Caballol, Nuria	Consorci Sanitari Integral, Hospital Moisés Broggi, Sant Joan Despí, Barcelona, Spain.	Site investigator/PI	Coordination at the center<br>Evaluation of participants and/or data<br>management
Cámara Lorenzo, Ana	Hospital Clínic de Barcelona, Barcelona, Spain	Site investigator	Nurse study coordinator
Canfield Medina, Héctor	Complejo Hospitalario Universitario de Ferrol<br>(CHUF), Ferrol, A Coruña, Spain	Site investigator	Evaluation of participants and/or data<br>management
Carrillo, Fátima	Hospital Universitario Virgen del Rocío, Sevilla,<br>Spain	Site investigator	Evaluation of participants and/or data<br>management
Carrillo Padilla, Francisco José	Hospital Universitario de Canarias, San Cristóbal de la Laguna, Santa Cruz de Tenerife,<br>Spain	Site investigator/PI	Coordination at the center<br>Evaluation of participants and/or data<br>management
Casas, Elena	Complejo Asistencial Universitario de Burgos,<br>Burgos, Spain	Site investigator	Evaluation of participants and/or data<br>management
Catalán, Maria José	Hospital Universitario Clínico San Carlos, Madrid, Spain	Site investigator/PI	Coordination at the center<br>Evaluation of participants and/or data<br>management
Clavero, Pedro	Complejo Hospitalario de Navarra, Pamplona,<br>Spain	Site investigator	Evaluation of participants and/or data<br>management
Cortina Fernández, A	Complejo Hospitalario Universitario de Ferrol<br>(CHUF), Ferrol, A Coruña, Spain	Site investigator	Coordination of blood extractions

Cosgaya, Marina	Hospital Clínic de Barcelona, Barcelona, Spain	Site investigator	Evaluation of participants and/or data management
Cots Foraster, Anna	Institut d'Assistència Sanitària (IAS)—Instituí Cátala de la Salud. Girona, Spain	Site investigator	Evaluation of participants and/or data management
Crespo Cuevas, Ane	Hospital del Mar, Barcelona, Spain.	Site investigator	Evaluation of participants and/or data management
Cubo, Esther	Complejo Asistencial Universitario de Burgos, Burgos, Spain	Site investigator/PI	Coordination at the center Evaluation of participants and/or data management
De Deus Fonticoba, Teresa	Complejo Hospitalario Universitario de Ferrol (CHUF), Ferrol, A Coruña, Spain	Site investigator	Nurse study coordinator Evaluation of participants and/or data management
De Fábregues-Boixar, Oriol	Hospital Universitario Vall d´Hebron, Barcelona, Spain	Site investigator/PI	Coordination at the center Evaluation of participants and/or data management
Díez Fairen, M	Hospital Universitari Mutua de Terrassa, Terrassa, Barcelona, Spain	Site investigator	Evaluation of participants and/or data management
Dotor García-Soto, Julio	Hospital Universitario Virgen Macarena, Sevilla, Spain	Site investigator/PI	Evaluation of participants and/or data management
Erro, Elena	Complejo Hospitalario de Navarra, Pamplona, Spain	Site investigator	Evaluation of participants and/or data management
Escalante, Sonia	Hospital de Tortosa Verge de la Cinta (HTVC), Tortosa, Tarragona, Spain	Site investigator/PI	Coordination at the center Evaluation of participants and/or data management
Estelrich Peyret, Elena	Institut d'Assistència Sanitària (IAS)—Instituí Cátala de la Salud. Girona, Spain	Site investigator	Evaluation of participants and/or data management
Fernández Guillán, Noelia	Complejo Hospitalario Universitario de Ferrol (CHUF), Ferrol, A Coruña, Spain	Site investigator	Neuroimaging studies
Gámez, Pedro	Complejo Asistencial Universitario de Burgos, Burgos, Spain	Site investigator	Evaluation of participants and/or data management
Gallego, Mercedes	Hospital La Princesa, Madrid, Spain	Site investigator	Evaluation of participants and/or data management
García Caldentey, Juan	Centro Neurológico Oms 42, Palma de Mallorca, Spain	Site investigator/PI	Coordination at the center Evaluation of participants and/or data management
García Campos, Cristina	Hospital Universitario Virgen Macarena, Sevilla, Spain	Site investigator	Evaluation of participants and/or data management
García Díez, Cristina	Complejo Hospitalario Universitario de Pontevedra (CHOP), Pontevedra, Spain	Site investigator (from May 2022)	neuropsychologist; evaluation of participants

García Moreno, Jose Manuel	Hospital Universitario Virgen Macarena, Sevilla, Spain	Site investigator/PI (until MAR/21)	Coordination at the center<br>Evaluation of participants and/or data management
Gastón, Itziar	Complejo Hospitalario de Navarra, Pamplona, Spain	Site investigator/PI	Coordination at the center<br>Evaluation of participants and/or data management
Gómez Garre, María del Pilar	Hospital Universitario Virgen del Rocío, Sevilla, Spain	Site investigator	Genetic studies coordination
Gómez Mayordomo, Víctor	Hospital Clínico San Carlos, Madrid, Spain	Site investigator	Evaluation of participants and/or data management
González Aloy, Javier	Institut d'Assistència Sanitària (IAS)—Instituí Cátala de la Salud. Girona, Spain	Site investigator	Evaluation of participants and/or data management
González Aramburu, Isabel	Hospital Universitario Marqués de Valdecilla, Santander, Spain	Site investigator	Evaluation of participants and/or data management
González Ardura, Jessica	Hospital Universitario Lucus Augusti (HULA), Lugo, Spain	Site investigator/PI (until FEB/21)	Evaluation of participants and/or data management
González García, Beatriz	Hospital La Princesa, Madrid, Spain	Site investigator	Nurse study coordinator
González Palmás, Maria Josefa	Complejo Hospitalario Universitario de Pontevedra (CHOP), Pontevedra, Spain	Site investigator	Evaluation of participants and/or data management
González Toledo, Gabriel Ricardo	Hospital Universitario de Canarias, San Cristóbal de la Laguna, Santa Cruz de Tenerife, Spain	Site investigator	Evaluation of participants and/or data management
Golpe Díaz, Ana	Complejo Hospitalario Universitario de Ferrol (CHUF), Ferrol, A Coruña, Spain	Site investigator	Laboratory analysis coordination
Grau Solá, Mireia	Consorci Sanitari Integral, Hospital Moisés Broggi, Sant Joan Despí, Barcelona, Spain	Site investigator	Evaluation of participants and/or data management
Guardia, Gemma	Hospital Universitari Mutua de Terrassa, Terrassa, Barcelona, Spain	Site investigator	Evaluation of participants and/or data management
Hernández Vara, Jorge	Hospital Universitario Vall d´Hebron, Barcelona, Spain	Site investigator/PI	Coordination at the center<br>Evaluation of participants and/or data management
Horta Barba, Andrea	Hospital de Sant Pau, Barcelona, Spain	Site investigator	Neuropsychologist; evaluation of participants
Idoate Calderón, Daniel	Complejo Hospitalario Universitario de Pontevedra (CHOP), Pontevedra, Spain	Site investigator (until May 2022)	neuropsychologist; evaluation of participants
Infante, Jon	Hospital Universitario Marqués de Valdecilla, Santander, Spain	Site investigator/PI	Coordination at the center<br>Evaluation of participants and/or data management
Jesús, Silvia	Hospital Universitario Virgen del Rocío, Sevilla, Spain	Site investigator	Evaluation of participants and/or data management

Kulisevsky, Jaime	Hospital de Sant Pau, Barcelona, Spain	Site investigator/PI	Coordination at the center<br>Evaluation of participants and/or data<br>management
Kurtis, Mónica	Hospital Ruber Internacional, Madrid, Spain	Site investigator/PI	Coordination at the center<br>Evaluation of participants and/or data<br>management
Labandeira, Carmen	Hospital Álvaro Cunqueiro, Complejo Hospitalario Universitario de Vigo (CHUVI),<br>Vigo, Spain	Site investigator	Evaluation of participants and/or data<br>management
Labrador Espinosa, Miguel Ángel	Hospital Universitario Virgen del Rocío, Sevilla,<br>Spain	Site investigator	Neuroimaging data analysis
Lacruz, Francisco	Complejo Hospitalario de Navarra, Pamplona,<br>Spain	Site investigator	Evaluation of participants and/or data<br>management
Lage Castro, Melva	Complejo Hospitalario Universitario de Pontevedra (CHOP), Pontevedra, Spain	Site investigator	Evaluation of participants and/or data<br>management
Lastres Gómez, Sonia	Complejo Hospitalario Universitario de Pontevedra (CHOP), Pontevedra, Spain	Site investigator	Neuropsychologist; evaluation of participants
Legarda, Inés	Hospital Universitario Son Espases, Palma de<br>Mallorca, Spain	Site investigator/PI	Coordination at the center<br>Evaluation of participants and/or data<br>management
López Ariztegui, Nuria	Complejo Hospitalario de Toledo, Toledo, Spain	Site investigator/PI	Evaluation of participants and/or data<br>management
López Díaz, Luis Manuel	Hospital Da Costa de Burela, Lugo, Spain	Site investigator	Evaluation of participants and/or data<br>management
López Domínguez, Daniel	Institut d'Assistència Sanitària (IAS)—Instituí<br>Cátala de la Salud. Girona, Spain	Site investigator	Evaluation of participants and/or data<br>management
López Manzanares, Lydia	Hospital La Princesa, Madrid, Spain	Site investigator/PI	Coordination at the center<br>Evaluation of participants and/or data<br>management
López Seoane, Balbino	Complejo Hospitalario Universitario de Ferrol<br>(CHUF), Ferrol, A Coruña, Spain	Site investigator	Neuroimaging studies
Lucas del Pozo, Sara	Hospital Universitario Vall d´Hebron, Barcelona, Spain	Site investigator	Evaluation of participants and/or data<br>management
Macías, Yolanda	Fundación Hospital de Alcorcón, Madrid, Spain	Site investigator	Evaluation of participants and/or data<br>management
Mata, Marina	Hospital Infanta Sofía, Madrid, Spain	Site investigator	Evaluation of participants and/or data<br>management
Martí Andres, Gloria	Hospital Universitario Vall d´Hebron, Barcelona, Spain	Site investigator	Evaluation of participants and/or data<br>management
Martí, Maria José	Hospital Clínic de Barcelona, Barcelona, Spain	Site investigator/PI	Coordination at the center<br>Evaluation of participants and/or

			data management
Martínez Castrillo, Juan Carlos	Hospital Universitario Ramón y Cajal, Madrid, Spain	Site investigator /PI	Coordination at the center Evaluation of participants and/or data management
Martinez-Martin, Pablo	Centro Nacional de Epidemiología y CIBERNED, Instituto de Salud Carlos III. Madrid	Collaborator in statistical and methods analysis	Methods and statistical reviewer
McAfee, Darrian	University of Pennsylvania, Philadelphia	Collaborator of English style	English style reviewer
Meitín, Maria Teresa	Hospital Da Costa de Burela, Lugo, Spain	Site investigator	Evaluation of participants and/or data management
Menéndez González, Manuel	Hospital Universitario Central de Asturias, Oviedo, Spain	Site investigator/PI	Coordination at the center Evaluation of participants and/or data management
Méndez del Barrio, Carlota	Hospital Universitario Virgen del Rocío, Sevilla, Spain	Site investigator	Evaluation of participants and/or data management
Mendoza Plasencia, Zebenzui	Hospital Universitario de Canarias, San Cristóbal de la Laguna, Santa Cruz de Tenerife, Spain	Site investigator	Evaluation of participants and/or data management
Mir, Pablo	Hospital Universitario Virgen del Rocío, Sevilla, Spain	Site investigator/PI	Coordination at the center Evaluation of participants and/or data management
Miranda Santiago, Javier	Complejo Asistencial Universitario de Burgos, Burgos, Spain	Site investigator	Evaluation of participants and/or data management
Morales Casado, Maria Isabel	Complejo Hospitalario de Toledo, Toledo, Spain.	Site investigator	Evaluation of participants and/or data management
Moreno Diéguez, Antonio	Complejo Hospitalario Universitario de Ferrol (CHUF), Ferrol, A Coruña, Spain	Site investigator	Neuroimaging studies
Nogueira, Víctor	Hospital Da Costa de Burela, Lugo, Spain	Site investigator/PI	Coordination at the center Evaluation of participants and/or data management
Novo Amado, Alba	Complejo Hospitalario Universitario de Ferrol (CHUF), Ferrol, A Coruña, Spain	Site investigator	Neuroimaging studies
Novo Ponte, Sabela	Hospital Universitario Puerta de Hierro, Madrid, Spain.	Site investigator	Evaluation of participants and/or data management
Ordás, Carlos	Hospital Rey Juan Carlos, Madrid, Spain, Madrid, Spain.	Site Investigator	Evaluation of participants and/or data management
Pagonabarraga, Javier	Hospital de Sant Pau, Barcelona, Spain	Site investigator	Evaluation of participants and/or data management

Pareés, Isabel	Hospital Ruber Internacional, Madrid, Spain	Site investigator	Evaluation of participants and/or data management
Pascual-Sedano, Berta	Hospital de Sant Pau, Barcelona, Spain	Site Investigator	Evaluation of participants and/or data management
Pastor, Pau	Hospital Universitari Mutua de Terrassa, Terrassa, Barcelona, Spain	Site investigator	Evaluation of participants and/or data management
Pérez Fuertes, Aída	Complejo Hospitalario Universitario de Ferrol (CHUF), Ferrol, A Coruña, Spain	Site investigator	Blood analysis
Pérez Noguera, Rafael	Hospital Universitario Virgen Macarena, Sevilla, Spain	Site investigator	Evaluation of participants and/or data management
Planas-Ballvé, Ana	Consorci Sanitari Integral, Hospital Moisés Broggi, Sant Joan Despí, Barcelona, Spain	Site investigator	Evaluation of participants and/or data management
Planellas, Lluís	Hospital Clínic de Barcelona, Barcelona, Spain	Site investigator (until DEC/19)	Evaluation of participants and/or data management
Prats, Marian Ángeles	Institut d'Assistència Sanitària (IAS)—Instituí Cátala de la Salud. Girona, Spain	Site investigator	Evaluation of participants and/or data management
Prieto Jurczynska, Cristina	Hospital Rey Juan Carlos, Madrid, Spain, Madrid, Spain	Site investigator/PI	Coordination at the center<br>Evaluation of participants and/or data management
Puente, Víctor	Hospital del Mar, Barcelona, Spain	Site investigator/PI	Coordination at the center<br>Evaluation of participants and/or data management
Pueyo Morlans, Mercedes	Hospital Universitario de Canarias, San Cristóbal de la Laguna, Santa Cruz de Tenerife, Spain	Site investigator	Evaluation of participants and/or data management
Puig Daví, Arnau	Hospital de Sant Pau, Barcelona, Spain	Site investigator	Evaluation of participants and/or data management
Redondo, Nuria	Hospital La Princesa, Madrid, Spain	Site Investigator	Evaluation of participants and/or data management
Rodríguez Méndez, Luisa	Complejo Hospitalario Universitario de Ferrol (CHUF), Ferrol, A Coruña, Spain	Site investigator	Blood analysis
Rodríguez Pérez, Amparo Belén	Hospital General Universitario de Elche, Elche, Spain	Site investigator	Evaluation of participants and/or data management
Roldán, Florinda	Hospital Universitario Virgen del Rocío, Sevilla, Spain	Site investigator	Neuroimaging studies
Ruíz de Arcos, María	Hospital Universitario Virgen Macarena, Sevilla, Spain.	Site investigator	Evaluation of participants and/or data management
Ruíz Martínez, Javier	Hospital Universitario Donostia, San Sebastián, Spain	Site investigator	Evaluation of participants and/or data management

Sánchez Alonso, Pilar	Hospital Universitario Puerta de Hierro, Madrid, Spain	Site investigator	Evaluation of participants and/or data management
Sánchez-Carpintero, Macarena	Complejo Hospitalario Universitario de Ferrol (CHUF), Ferrol, A Coruña, Spain	Site investigator	Neuroimaging studies
Sánchez Díez, Gema	Hospital Universitario Ramón y Cajal, Madrid, Spain	Site investigator	Evaluation of participants and/or data management
Sánchez Rodríguez, Antonio	Hospital Universitario Marqués de Valdecilla, Santander, Spain	Site investigator	Evaluation of participants and/or data management
Santacruz, Pilar	Hospital Clínic de Barcelona, Barcelona, Spain	Site investigator	Evaluation of participants and/or data management
Santos García, Diego	CHUAC, Complejo Hospitalario Universitario de A Coruña	Coordinator of the Project	Coordination of the COPPADIS-2015
Segundo Rodríguez, José Clemente	Complejo Hospitalario de Toledo, Toledo, Spain	Site investigator	Evaluation of participants and/or data management
Seijo, Manuel	Complejo Hospitalario Universitario de Pontevedra (CHOP), Pontevedra, Spain	Site investigator/PI	Coordination at the center Evaluation of participants and/or data management
Sierra, María	Hospital Universitario Marqués de Valdecilla, Santander, Spain	Site investigator	Evaluation of participants and/or data management
Solano, Berta	Institut d'Assistència Sanitària (IAS)—Instituí Cátala de la Salud. Girona, Spain	Site investigator/PI	Coordination at the center Evaluation of participants and/or data management
Suárez Castro, Ester	Complejo Hospitalario Universitario de Ferrol (CHUF), Ferrol, A Coruña, Spain	Site investigator	Evaluation of participants and/or data management
Tartari, Juan Pablo	Hospital Universitari Mutua de Terrassa, Terrassa, Barcelona, Spain	Site investigator	Evaluation of participants and/or data management
Valero, Caridad	Hospital Arnau de Vilanova, Valencia, Spain	Site investigator	Evaluation of participants and/or data management
Vargas, Laura	Hospital Universitario Virgen del Rocío, Sevilla, Spain	Site investigator	Evaluation of participants and/or data management
Vela, Lydia	Fundación Hospital de Alcorcón, Madrid, Spain	Site investigator/PI	Coordination at the center Evaluation of participants and/or data management
Villanueva, Clara	Hospital Universitario Clínico San Carlos, Madrid, Spain	Site investigator	Evaluation of participants and/or data management
Vives, Bárbara	Hospital Universitario Son Espases, Palma de Mallorca, Spain	Site investigator	Evaluation of participants and/or data management
